# The Effect of Hemodynamic Remodeling on the Survival of Arterialized Venous Flaps

**DOI:** 10.1371/journal.pone.0079608

**Published:** 2013-11-12

**Authors:** Hede Yan, Jon Kolkin, Bin Zhao, Zhefeng Li, Shichao Jiang, Wei Wang, Zhen Xia, Cunyi Fan

**Affiliations:** 1 Department of Orthopedics, The Sixth Affiliated People’s Hospital, Shanghai Jiao Tong University, Shanghai, China; 2 Department of Orthopaedics (Division of Plastic and Hand Surgery), The Second Affiliated Hospital of Wenzhou Medical College, Wenzhou, China; 3 Department of Plastic and Hand Surgery, Duke Raleigh Hospital, Raleigh, North Carolina, United States of America; University Medical Center Utrecht, Netherlands

## Abstract

**Objective:**

To evaluate the effect of hemodynamic remodeling on the survival status of the arterialized venous flaps (AVFs) and investigate the mechanism of this procedure.

**Materials and Methods:**

Two 7 x 9 cm skin flaps in each rabbit (n=36) were designed symmetrically in the abdomen. The thoracoepigastric pedicle and one femoral artery were used as vascular sources. Four groups were included: Composite skin grafts group and arterial perfusion group were designed in one rabbit; AVF group and hemodynamic remodeling group by ligation of the thoracoepigastric vein in the middle were outlined in another rabbit. Flap viability, status of vascular perfusion and microvasculature, levels of epidermal metabolite and water content in each group were assessed.

**Results:**

Highly congested veins and simple trunk veins were found using angiography in the AVF group; while a fairly uniform staining and plenty of small vessels were observed in the hemodynamic remodeling group. The metabolite levels of the remodeling group are comparable with those in the arterial perfusion group. There was no statistically significant difference in the percentage of flap survival between the arterial perfusion group and hemodynamic remodeling group; however, significant difference was seen between the AVF group and the hemodynamic remodeling group.

**Conclusions:**

Under the integrated perfusion mode, the AVFs are in an over-perfusion and non-physiological hemodynamic state, resulting in unreliability and unpredictability in flap survival; under the separated perfusion mode produced by remodeling, a physiological-like circulation will be created and therefore, better flap survival can be expected.

## Introduction

Great success has been achieved in conventional reconstructive surgery since the advent of microsurgical techniques; however, a variety of problems still cannot be satisfactorily addressed using physiological flaps[1]. In recent years, the arterialized venous flap (AVF) is highly regarded in microsurgical and reconstructive surgeries based on advantages of ease of design and harvest without the need to perform deep dissection, no sacrifice of a major artery at the donor site, no limitation of the donor sites, and less donor-site morbidity and being non- bulky[2]. In particular, it has been gradually popularized in the reconstruction of hand and digit injuries in selected cases [3,4]. 

The AVF was first investigated using a rat model in 1981[5] and shortly after, the first clinical series of AVFs was reported in 1987 [6]. Since then, a variety of studies have been carried out[7-15]. Nonetheless, the application of AVFs has still been limited owing to the unpredictability of published survival rates[4,16-18].. In our recent systematic review[2], congestion rates occurred up to 100% in certain series and a partial necrosis rate and total necrosis rate were 42.5% and 7.5%, respectively. These findings indicate that the application of AVFs should be cautious in practice.

The reason for flap failure of AVFs remains unclear, making it difficult or even impossible to reliably prevent future flap failures[19]. The ongoing uncertainty regarding exact mechanisms of venous flap perfusion has been considered as the bottleneck problem for its survival[20-22]. However, as a non-physiological flap with its circulation solely based on venous system, the hemodynamic features of this flap definitely play a key role in this situation. These issues have not been fully understood and controversies still exist in this field[23-25]. Based on the understanding of the hemodynamic characteristics of AVFs, we hypothesize that its hemodynamic feature is characterized as an integrated perfusion mode, resulting in an over-perfusion state and unreliable survival; after hemodynamic remodeling creating a separated perfusion mode, a non-physiological flap then turns into a physiological-like perfusion flap, thus achieving reliable survival. In the present study, we attempt to verify our hypothesis and further investigate the possible mechanism of this technique.

## Materials and Methods

### Animal models and grouping

Adult white Japanese rabbits of both sexes, weighing between 3.5 and 4 kg, were used. All experiments were performed according to ethical approval (Institutional Animal Care and Use Committee, Shanghai Jiaotong University School of Medicine) and the National Research Council’s guidelines for the care and use of laboratory animals were followed. The rabbits were anesthetized with an ear vein injection of choral hydrate (10%) at a dose of 1.5ml/kg, and the abdominal area of the rabbit was then carefully shaved. During the several-hour operation, the animals were maintained under anesthesia by re-injection of choral hydrate.

**Figure 1:**
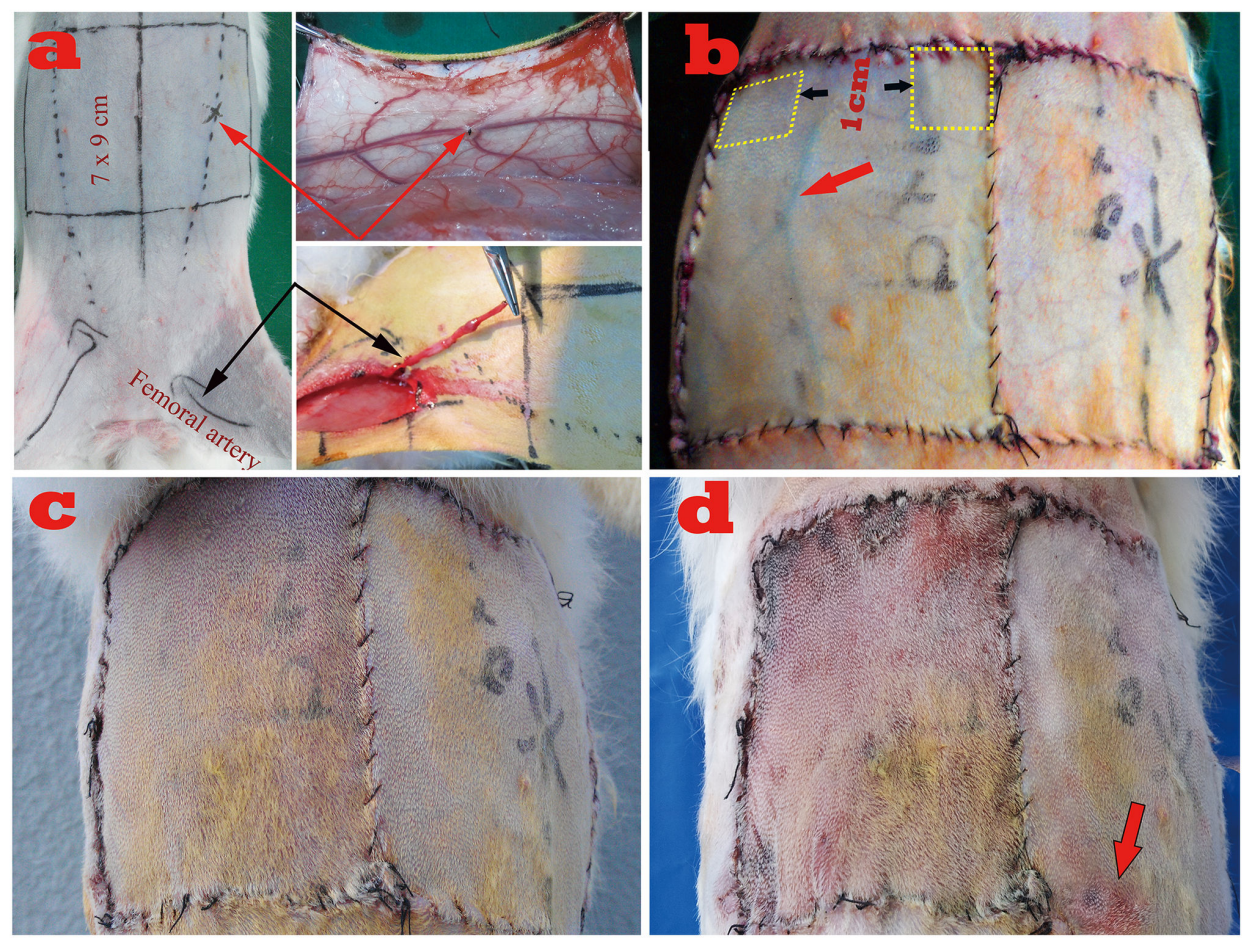
Flap design and postoperative appearance. a. Flap design: demonstration of flap location and vascular pedicles; the red arrows on the top show the ligation site; the black arrows at the bottom show the proximally transferred femoral artery. b. Postoperative view shortly after flap transfer: left, the flap of AVF group, showing a significantly engorged thoracoepigastric vein (red arrow); the yellow boxes indicate the sites of specimen harvested in every group; right, the flap appearance of HR group with no signs of venous congestion. c. Postoperative view 3 days after surgery: left, flap appearance of AVF group with conspicuous sign of venous congestion; right, flap appearance of HR group with no signs of venous congestion. d. Postoperative view 7 days after surgery: left, partial flap loss in AVF group; right, only marginal flap loss in HR group (red arrow).

Thirty-six rabbits were randomly divided into two groups using MS Excel software (n=18) and two subgroups were further assigned in each group. Two 7 x 9 cm (width x length) skin flaps in each rabbit were designed symmetrically in the abdomen, with the proximal border just below the level of the first nipple (Figure 1a). The flap was elevated including the epidermis, dermis, subcutaneous tissue, and the panniculus carnosis. Control groups I and II were randomly designed on either side of the abdomen of the same rabbit, respectively: in control group I (composite skin grafts group), the flaps were freed and laid back as composite grafts; in control group II (arterial perfusion group), the thoracoepigastric artery and vein were maintained as a physiological flap. Control group III and hemodynamic remodeling group (HR group) were randomly tailored in the same rabbit using either side of the abdomen, respectively: in control group III (arterialized venous perfusion group, AVF group), the ipsilateral femoral artery was dissected and reversed then anastomosed with the distal end of thoracoepigastric vein using 11-0 sutures, simply leaving the proximal thoracoepigastric vein intact to provide venous outflow. The flaps were freed completely and then sutured back in situ; in HR group, the flaps were first elevated in a similar manner as AVF group, but the thoracoepigastric vein was ligated with a suture in the middle. (Figure 1 b) 

**Figure 2:**
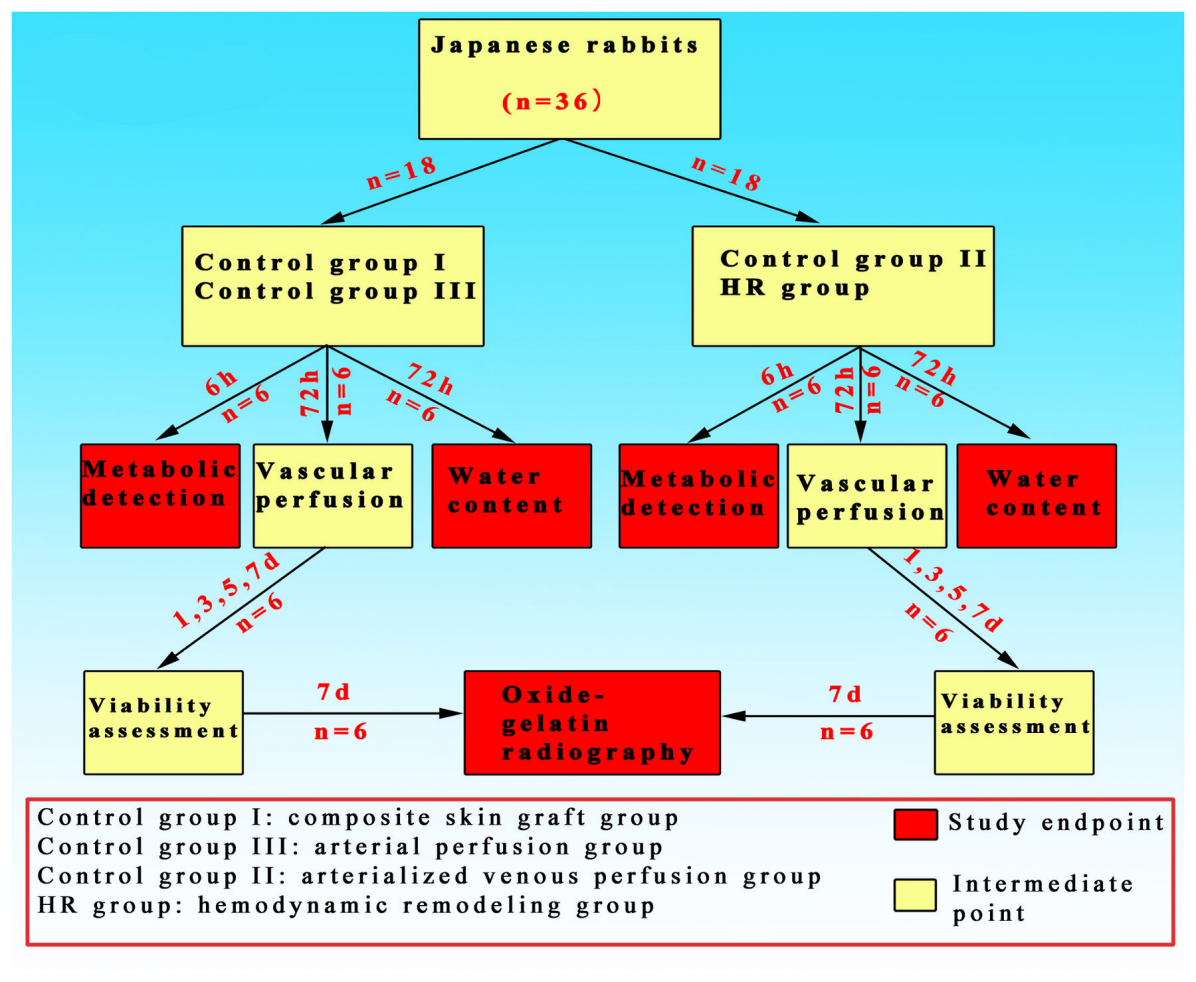
Flowchart of rabbits handling at different study endpoints.

All surgical procedures were performed by the same surgical team aseptically. A soft padded dressing was used to cover the flap in order to guard against pressure when the rabbit was lying down. Analgesia and antibiotics were administered for three days postoperatively. At each study endpoints, the patency of the inflow and outflow vessels was checked in all the rabbits. Only those with confirmed patency were included and those with occlusion were replaced accordingly. The detailed animal handling procedures at different study endpoints were shown in Figure 2.

## Detection of Epidermal Metabolite Levels

The epidermal metabolite levels of glucose and lactate were detected 6 hours postoperatively. Six rabbits were randomly selected in each group. While the rabbits were still alive and anesthetized, two specimens of the same size (1x 1 cm) were obtained from the proximal portion of each flap.(Figure 1a) The specimens were then immediately frozen in liquid nitrogen and grinded into 10% tissue homogenate. The total protein content of the tissue lysate samples was determined using the Bradford assay [26]. The contents of glucose and lactate were measured by colorimetric assay kits (Nanjing Jiancheng Bioengineering Institute, Jiangsu, China) based on the following equation: content of lactate or glucose (mmol / gprot )= [(OD of the sample tube - OD of blank tube) / (OD of standard tube-OD of standard blank tube)] × concentration of standard sample / total protein content.

### Determination of vascular perfusion

Six rabbits in each group were selected randomly 72 hours after surgery. Fluorescein (75 mg/kg, Sigma) in a 5% solution [27]was injected through an ear vein. Then ultraviolet pictures, under a fluorescent light, were obtained using a Canon camera. The fluorescent staining was recorded at 30 minutes and finally 60 minutes after injection. The staining area was quantified based on standardized color pictures using a computerized image analysis system (Image Pro Plus 5.0, Media Cybernetics, Inc., Bethesda, USA)[28].

### Water content analysis in flaps

Another six rabbits were randomly selected in each group 72 hours after surgery. The flap samples (1 x1 cm^2^) as shown in Figure 1 were harvested and dried with a stencil for analysis and the wet weight of the sample was measured with precision electronic scale. Then the flap with known wet weight was placed in a drying oven and kept for 24 hours at 65°C. Afterward, the dry weight of the flap was measured using the same precision scale. The formula for calculating the water content [29]:

Water content (%) = (Wet weight-Dry weight)/Wet weight × 100

### Viability assessment

Flap viability was visually assessed on postoperative days 1, 3, 5 and 7. Viability was determined by flap color, texture, degree of swelling, hair growth, and the presence of necrosis. Quantification of survival was expressed as a percentage of the total flap surface area. From standardized color pictures, total flap surface areas and necrotic areas were measured with the same analysis system used for the detection of fluorescent staining. The same investigator evaluated the flaps to minimize inter-observer bias and variability. 

### Microvasculature examination of functional vessels

All rabbits in each group (n=6) underwent whole-body radiopaque contrast injection 7 days after flap transfer via the common carotid artery using a modified lead oxide–gelatin technique[30]. The injected rabbits were then kept in a refrigerator overnight for enhancement of peripheral diffusion and stabilization of the contrast medium. The flaps were then harvested and radiographed (FCR XG-1, Japan) at settings of 40 kV, 5 mA, with 0.1-s exposure. The X-ray data was then imported onto an image station directly through an FCR system. The average vessel density of the flap was calculated using Scion Image Beta 4.02 (Scion Corporation, Frederick, Md.) [30].

### Data collection and statistical analysis

All the defined outcome parameters were studied in a blinded fashion. Nonparametric method of Kruskal Wallis test was used for all the analyses with SPSS v. 19.0. Mann-Whiteney U test with Bonferroni correction was selected for the statistical analysis between groups. Data were expressed as mean ± standard deviations. 

## Results

The composite grafts in control group I gradually became necrotic 7 days postoperatively and the flaps in the arterial perfusion group all survived uneventfully; in the AVF group, congestion and swelling developed shortly after surgery and became increasingly significant 3 days postoperatively, all the flaps achieved only partial survival (Figure 1, b-c); in the HR group, mild congestion and swelling were visible on the first day and subsided by the third day. All the flaps in this group eventually survived with the occurrence of small marginal necrosis distally. (Figure 1 d) There was no statistically significant difference in the percentage of flap survival between the arterial group and the HR group (p=0.078); however, a significant difference was seen between the AVF group and the HR group (p=0.002). (Table 1).

**Table 1:** Results of flap survival.

Groups	Mean area of survival (cm2)	Mean survival percentage (survival area / Total flap, %)^▲^
Control group I (Composite grafts group)	5.41 ± 1.67	4.03 ± 1.57
Control group II (Arterial perfusion flap)	151.03 ± 3.47	99.37 ± 0.58
Control group III (Arterialized venous perfusion group)	87.48 ± 5.40	59.15 ± 4.23
Hemodynamic remodeling group	148.68 ± 4.41	97.67 ± 1.72

▲ There was no statistically significant difference in the percentage of flap survival between the arterial perfusion group and the prefabricated group (p > 0.05); however, when comparing these two groups with the other two groups, the differences were significant. (p<0.05)

The results of the epidermal content of glucose and lactate in each group were shown in Table 2. Significant differences in lactate and glucose levels were seen among these four groups (p<0.001); while Mann-Whitney test with Bonferroni correction showed no significant differences in the level of lactate (p=0.262) or glucose (p=0.229) between arterial perfusion group and HR group, respectively; however, significantly higher levels of lactate and lower levels of glucose were noted in AVF group than those in the HR group, respectively (both p=0.004).

**Table 2:** Epidermal metabolite levels 6 hours after surgery^**▲**^.

Groups	Glucose (mmol/gprot)	Lactate (mmol/gprot)	Lactate/Glucose Ratio
Control group I (Composite grafts group)	0.346±0.033	1.742±0.146	5.05±0.295
Control group II (Arterial perfusion group)	1.003±0.024	0.795±0.028	0.794±0.033
Control group III (Arterialized venous perfusion group)	0.572±0.019	1.58±0.164	2.77±0.38
Hemodynamic remodeling group	0.988±0.019	0.848±0.082	0.858±0.068

▲ Values of the control group I and III were significantly different from the other two groups (p<0.001); however, no differences were noted between the Hemodynamic remodeling group and control group II (P>0.05).

**Figure 3:**
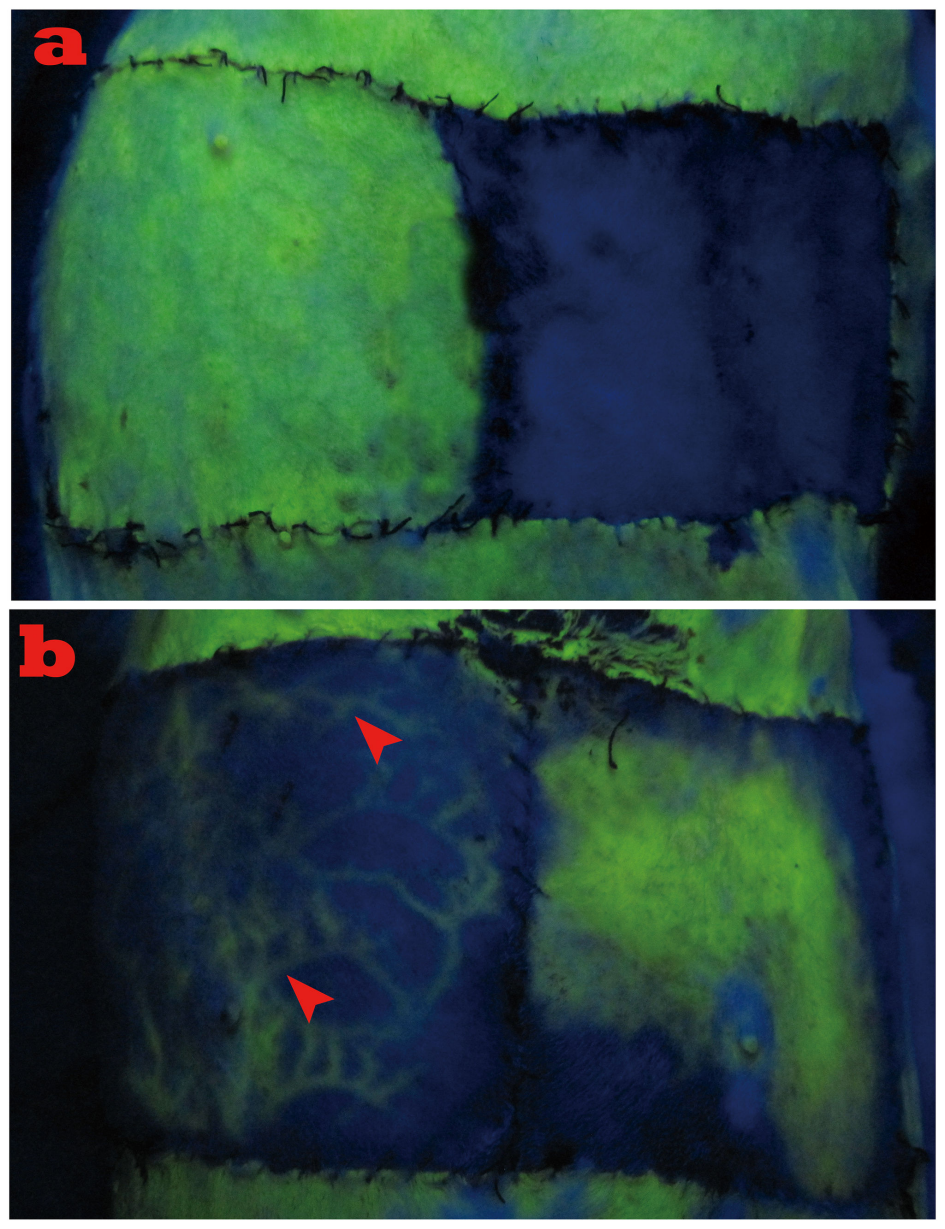
Results of fluorescent staining 72 hours after surgery. a. Left, bright fluorescence immediately after injection as opposed to no staining in the composite skin-grafting group (right). b. Left, plenty of engorged veins were scattered from distal to proximal portion of the flap with no signs of uniform distribution of fluorescein staining (solid arrow heads indicate the engorged veins); right, fluorescein staining was well-distributed around most portion of the flap in the HR group.

Fluorescent staining revealed that the entire flap in the arterial perfusion group (Figure 3a, left) and normal skin around the flaps showed bright fluorescence immediately after injection as opposed to no staining in the control group I (Figure 3a, right). The flap in the HR group initially stained from the center of the flap and then the fluorescein staining was well-distributed around most portions of the flap within 30 to 60 minutes (Figure 3b, right), while no uniform distribution of fluorescein staining was observed in the AVF group. Surprisingly, plenty of engorged veins were scattered from the distal to proximal portion of the flap. (Figure 3b, left) Significant differences in staining areas were seen between the AVF group and the HR group (p<0.001).

**Figure 4:**
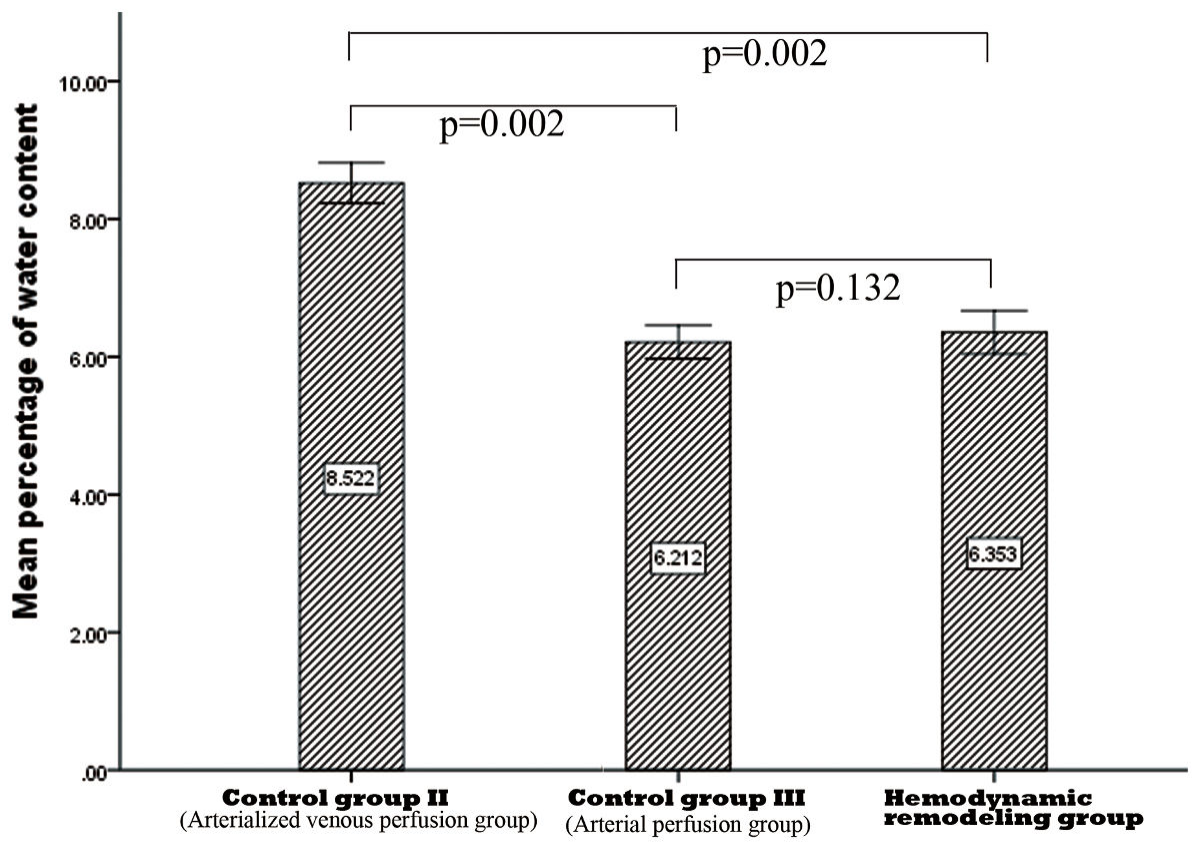
Results of water content of different groups 72 hours after surgery.

The average value of water content in the AFV group was the highest with 8.52 ± 0.15 and the difference was statistically significant in comparison with the arterial perfusion group and the HR group, respectively. The water content in the arterial perfusion group was slightly lower than that in the HR group; however, the difference was not statistically significant. (Figure 4)

**Figure 5:**
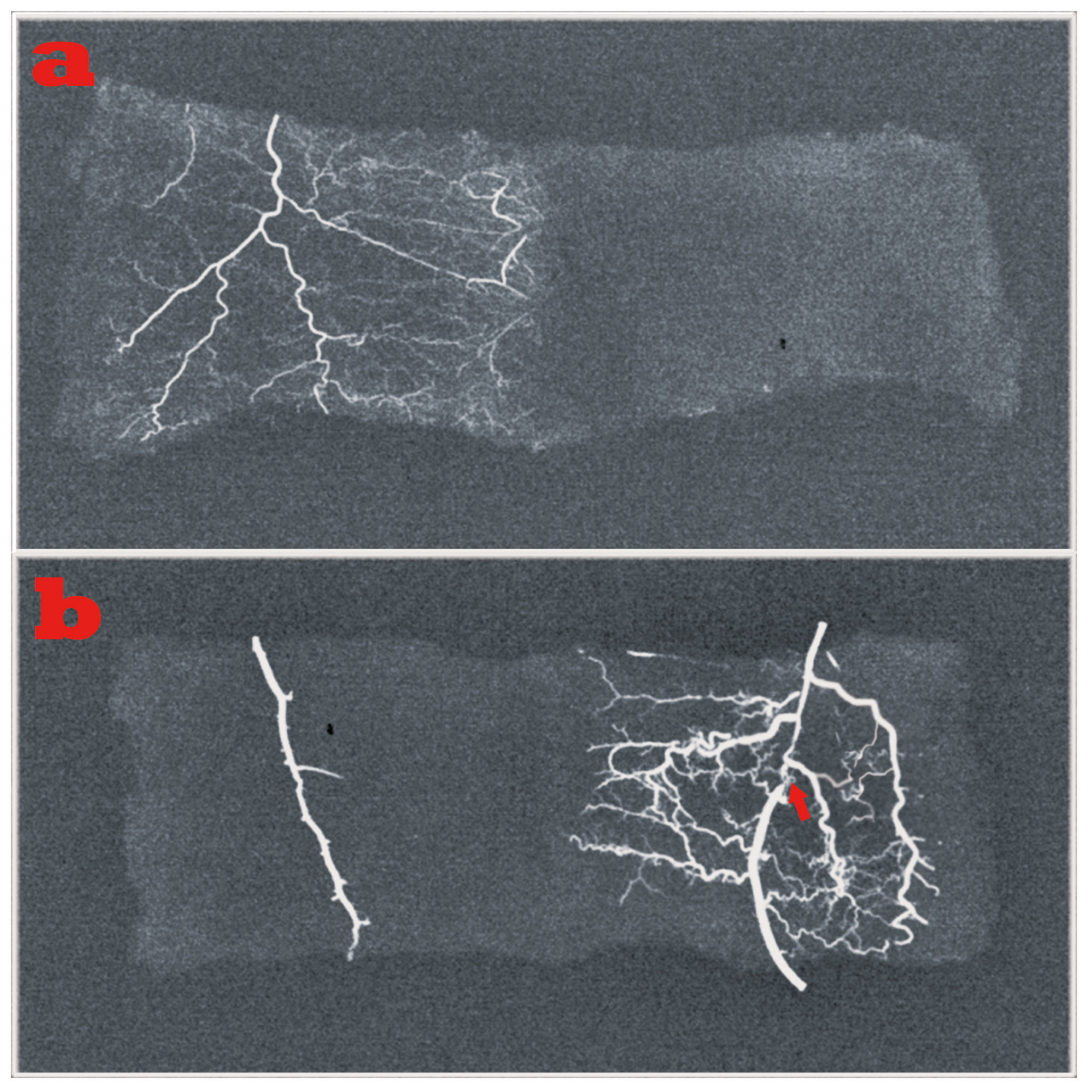
Microvasculature examination of functional vessels. a. Left, dense vessels were distributed regularly around the flaps of the arterial perfusion group; right, no vessels were visible in flaps of the composite skin-grafting group. b. Left, only a trunk vessel without small vessels was noted in the AVF group; right, plenty of small vessels as seen in the arterial perfusion group was visible in HR group ( the red arrow indicates the site of ligation).

Microvasculature examination of functional vessels showed that dense vessels were distributed regularly around the flaps in the arterial perfusion group (Figure 5 a, left), while no vessels were visible in control group I (Figure 5 a, right). Similar results as seen in the arterial perfusion group were also observed in the HR group. (Figure 5 b, right) In contrast, only a trunk vessel without small vessels was noted in the AVF group. (Figure 5 b, left). Significant differences in average vessel density within the flap were revealed between the HR group and AVF group (p<0.001); while no significant difference was seen between the HR group and the arterial perfusion group (p=0.471).

## Discussion

Venous flaps differ from conventional flaps in that the classic Harvesian model of arterial inflow-capillaries-venous outflow is replaced by the venous inflow-venous outflow[5]. There are considerable controversies on the real nature of their survivals accompanied with their advent. Possible mechanisms for the survival of the venous flaps are based on three main theories. These include “A-V shunting” or retrograde flow from the venous system to the arterial system via paralyzed arterial-venous shunts[31], “reverse flow” or a “to and fro” intermittent flow with perfusion of the blood from the flap vein to the capillary and then back to the vein[32], and finally “capillary bypass” or flow through the venous system without entrance into the arterial side until neovascularization[27]. However, no consensus regarding the exact mechanism for venous flap survival has been addressed, resulting in limited application in clinical settings.

In addition to perfection of the surgical technique and shortening the operating duration by experience, several strategies have also been attempted to improve the survival predictability of AVFs, such as using a relatively small afferent and relatively large efferent vein[33], anastomosing multiple draining veins[22], employing various surgical or chemical delay tactics[34,35] and pre-arterialization techniques[5], as well as pre-arterialization combined with A delay procedure[36]. Despite all these attempts, a reliable and predictable method of ensuring success in flap survival remains elusive; this is hardly surprising, as the root cause of venous congestion has not been effectively addressed[19]. All these procedures mainly focus on solving the resultant problems: venous congestion. 

Known as a non-physiological flap, the root cause of the AVF is derived from the features of its hemodynamics. Lam et al [19]postulated that because of the lower resistance in the efferent end of the flap, blood preferentially flows through the arterio-venous shunt instead of into the peripheries, thus bypassing the flap. In addition, the pressure within the efferent vein(s) effectively equilibrates with the afferent arterial pressure. If this pressure exceeds that of the peripheral tissue, blood cannot drain from the peripheral tissue with resultant venous congestion.

**Figure 6:**
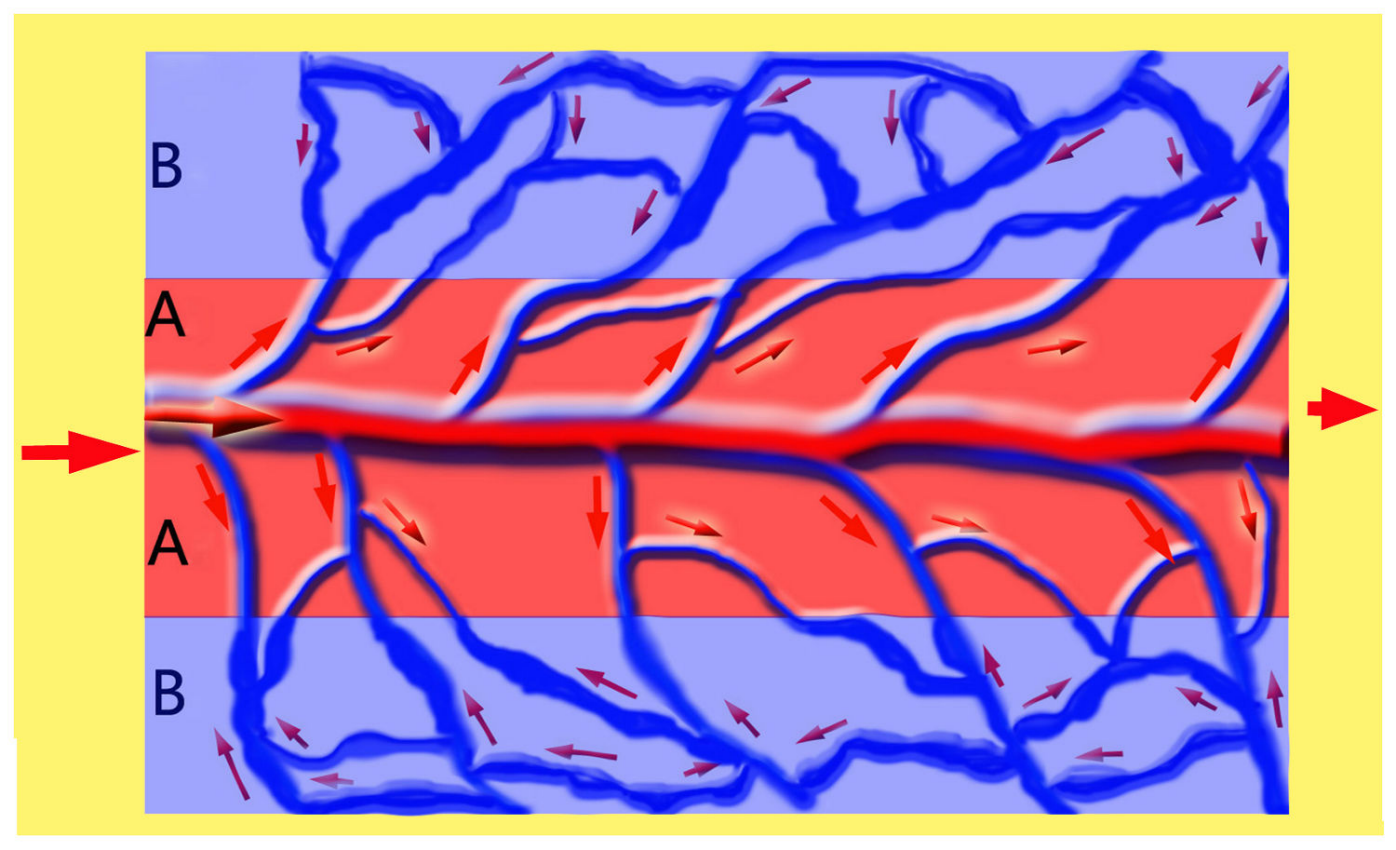
Demonstration of hemodynamic features of the AVF. An integrated perfusion mode, resulting in an over-perfusion state. Zone A indicates an area with a high pressure; zone B indicates an highly-congested area with disorders in circulation.

**Figure 7:**
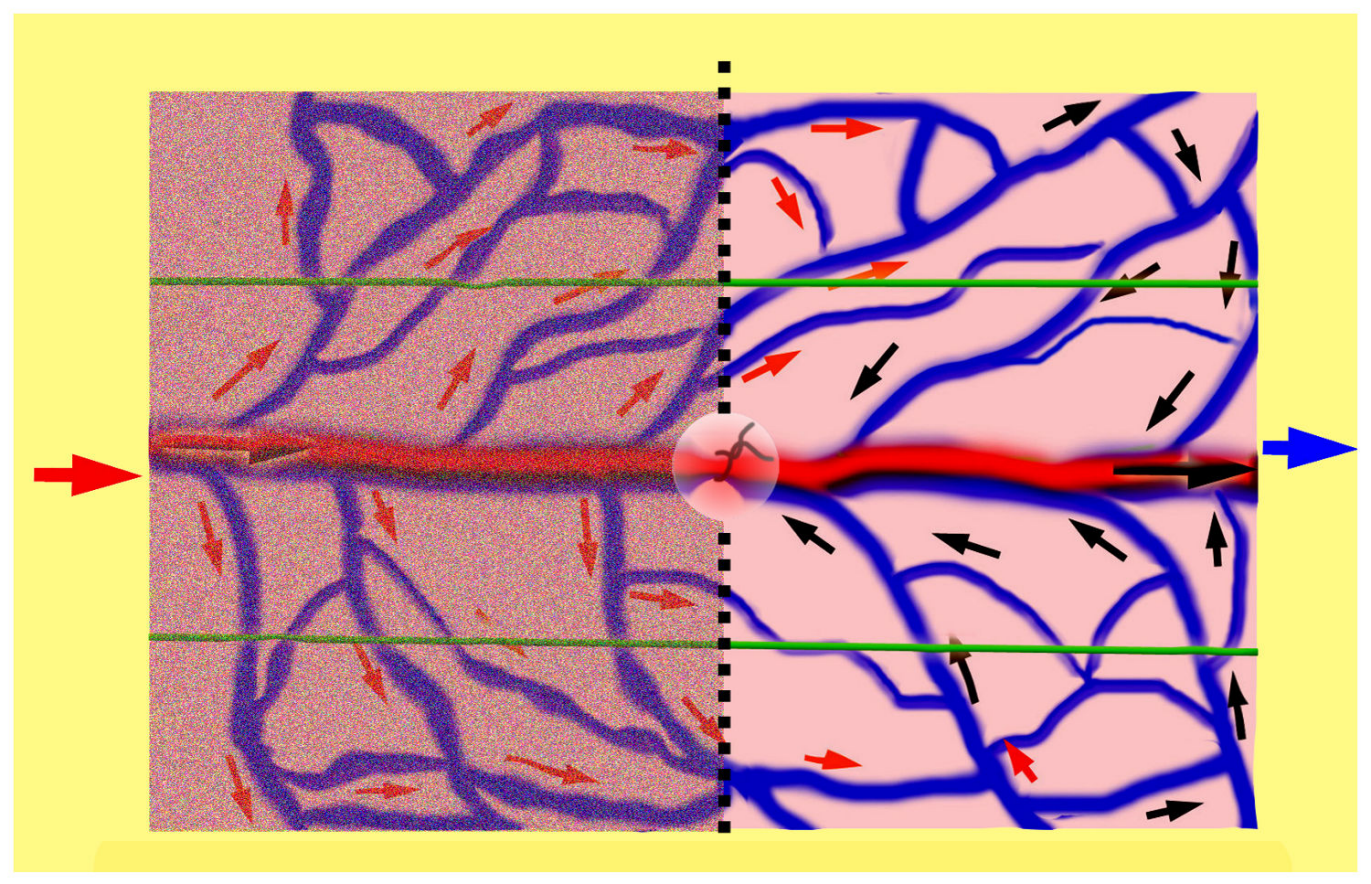
Demonstration of hemodynamic features after remodeling. Characterized with a separated perfusion mode, generating a physiological-like circulation pattern. The left half to the middle line is imagined as an inflow end and the right half as the outflow end; the circle in the center indicates the ligation of the target vein.

Therefore, as demonstrated in Figure 6, in a typical flow-through type of AVF, the flap can be imaginatively divided into two zones: the central zone A as a high-pressure area and the peripheral zone B as highly-congested area. In this flap, the hemodynamic feature is characterized as an integrated perfusion mode, resulting in an over-perfusion state. After hemodynamic remodeling as shown in Figure 7, the circulation pattern converts into a separated perfusion mode: the left half may be taken as an inflow zone and the right half as the outflow zone, thus a non-physiological flap, to some extent, turns into a physiological-like perfusion flap. In this way, the survival of AVFs are becoming reliable and predictable, because a flap can survive well without the classic Harvesian model of circulation as long as there is a constant and unidirectional flow of blood[37].

Our findings in this study provide solid evidence for this theory. Highly congested veins were found using fluorescent staining in the AVF group (control group III, Figure 3b, left); in contrast, a fairly uniform staining was seen after hemodynamic remodeling (Figure 3b, right). Furthermore, no small vessels were perfused with lead oxide–gelatin in the AVFs, indicating the flap was in an over-perfusion state with high pressure in the small vessels, which prevented the perfusion of the lead oxide–gelatin (Figure 5 b, left); while a relatively similar picture as found in the physiological flap (control group II, arterial perfusion flap) was obtained in the HR group (Figure 5 b, right). These results confirmed the efficiency of physiological remodeling in microvasculature. 

Further investigations revealed that the HR procedure had significant effects on the metabolism of the flaps: the AVFs sustained obvious edema with a higher percentage of water content in comparison with those undergoing the hemodynamic remodeling procedure. Moreover, when flaps suffer from ischemia, the glucose concentration will decrease dramatically and the lactate concentration will increase significantly with a much higher lactate-to-glucose (L/G) ratio, which is known to be a sensitive marker of ischemia and has been widely accepted for evaluation of tissue metabolic status[38-40]. In the present study, a comparable L/G ratio was obtained in the HR group and arterial perfusion group, which is significantly lower than those of the other two groups, respectively; the differences in lactate and glucose levels among groups also indicate that the metabolic level in the flaps after remodeling is comparable with that in the physiological flaps. The flap viability also verified the success of HR in AVFs with a survival percentage similar to that of conventional flaps.

In the literature, this procedure has been primarily introduced as a shunt-restricted technique. Lin et al [14] reported 15 cases using this technique with 100% survival rate and Lam et al[19] also initially presented their hypotheses regarding the possible mechanism of action for this procedure. Based on the radical changes on hemodynamics in the flaps undergoing this procedure, we used the word “hemodynamic remodeling” instead of “shunt-restricted” to better address this technique.

In conclusion, under the integrated perfusion mode, the AVFs are in an over-perfusion and non-physiological hemodynamic state, resulting in unreliability and unpredictability in flap survival; under the separated perfusion mode produced by remodeling, a physiological-like circulation will be created and therefore, better flap survival can be expected.

## References

[1] Tan BK, Chen HC, He TM, Song IC (2004) Flap prefabrication - the bridge between conventional flaps and tissue-engineered flaps. Ann Acad Med Singapore 33: 662-666. PubMed: 15536673. 15536673

[2] Yan H, Brooks D, Ladner R, Jackson WD, Gao W et al. (2010) Arterialized venous flaps: a review of the literature. Microsurgery 30: 472-478. doi:10.1002/micr.20769. PubMed: 20238385. 20238385

[3] Woo SH, Kim KC, Lee GJ, Ha SH, Kim KH et al. (2007) A retrospective analysis of 154 arterialized venous flaps for hand reconstruction: an 11-year experience. Plast Reconstr Surg 119: 1823-1838. doi:10.1097/01.prs.0000259094.68803.3d. PubMed: 17440363. 17440363

[4] Yan H, Zhang F, Akdemir O, Songcharoen S, Jones NI et al. (2011) Clinical applications of venous flaps in the reconstruction of hands and fingers. Arch Orthop Trauma Surg 131: 65-74. doi:10.1007/s00402-010-1107-2. PubMed: 20461524. 20461524

[5] Nakayama Y, Soeda S, Kasai Y (1981) Flaps nourished by arterial inflow through the venous system: an experimental investigation. Plast Reconstr Surg 67: 328-334. doi:10.1097/00006534-198103000-00009. PubMed: 7232566. 7232566

[6] Tsai TM, Matiko JD, Breidenbach W, Kutz JE (1987) Venous flaps in digital revascularization and replantation. J Reconstr Microsurg 3: 113-119. doi:10.1055/s-2007-1006973. PubMed: 3560036. 3560036

[7] Inoue G, Tamura Y (1991) One-stage repair of both skin and tendon digital defects using the arterialized venous flap with palmaris longus tendon. J Reconstr Microsurg 7: 339-343. doi:10.1055/s-2007-1006794. PubMed: 1753376. 1753376

[8] Inoue G, Maeda N (1988) Arterialized venous flap coverage for skin defects of the hand or foot. J Reconstr Microsurg 4: 259-266. doi:10.1055/s-2007-1006929. PubMed: 3172043. 3172043

[9] Cho BC, Byun JS, Baik BS (1999) Dorsalis pedis tendocutaneous delayed arterialized venous flap in hand reconstruction. Plast Reconstr Surg 104: 2138-2144. doi:10.1097/00006534-199912000-00030. PubMed: 11149781. 11149781

[10] Tang YB, Simchon S, Chen HC (2000) Microcirculation of a venous flap: an experimental study with microspheres in rabbits. Scand J Plast Reconstr Surg Hand Surg 34: 207-212. doi:10.1080/02844310050159774. PubMed: 11020916. 11020916

[11] Brooks D, Buntic R, Buncke HJ (2002) Use of a venous flap from an amputated part for salvage of an upper extremity injury. Ann Plast Surg 48: 189-192. doi:10.1097/00000637-200202000-00013. PubMed: 11910226. 11910226

[12] Hyza P, Vesely J, Stupka I, Cigna E, Monni N (2005) The bilobed arterialized venous free flap for simultaneous coverage of 2 separate defects of a digit. Ann Plast Surg 55: 679-683. doi:10.1097/01.sap.0000178806.37967.9f. PubMed: 16327475. 16327475

[13] Kong BS, Kim YJ, Suh YS, Jawa A, Nazzal A et al. (2008) Finger soft tissue reconstruction using arterialized venous free flaps having 2 parallel veins. J Hand Surg Am 33: 1802-1806. doi:10.1016/j.jhsa.2008.08.001. PubMed: 19084182. 19084182

[14] Lin YT, Henry SL, Lin CH, Lee HY, Lin WN et al. (2010) The shunt-restricted arterialized venous flap for hand/digit reconstruction: enhanced perfusion, decreased congestion, and improved reliability. J Trauma 69: 399-404. doi:10.1097/TA.0b013e3181bee6ad. PubMed: 20375918. 20375918

[15] Yan H, Gao W, Zhang F, Li Z, Chen X et al. (2012) A comparative study of finger pulp reconstruction using arterialised venous sensate flap and insensate flap from forearm. J Plast Reconstr Aesthet Surg 65: 1220-1226. doi:10.1016/j.bjps.2012.03.036. PubMed: 22583834. 22583834

[16] Chen HC, Tang YB, Noordhoff MS (1991) Four types of venous flaps for wound coverage: a clinical appraisal. J Trauma 31: 1286-1293. doi:10.1097/00005373-199109000-00014. PubMed: 1920561. 1920561

[17] Xiu ZF, Chen ZJ (1995) Clinical applications of venous flaps. Ann Plast Surg 34: 518-522. doi:10.1097/00000637-199505000-00011. PubMed: 7639490. 7639490

[18] Yilmaz M, Menderes A, Karataş O, Karaca C, Barutçu A (1996) Free arterialised venous forearm flaps for limb reconstruction. Br J Plast Surg 49: 396-400. doi:10.1016/S0007-1226(96)90009-0. PubMed: 8881787. 8881787

[19] Lam WL, Lin WN, Bell D, Higgins JP, Lin YT et al. (2013) The physiology, microcirculation and clinical application of the shunt-restricted arterialized venous flaps for the reconstruction of digital defects. J Hand Surg Eur Vol 38: 352-365. PubMed: 23186864. 10.1177/1753193412468632 23186864

[20] Moshammer HE, Schwarzl FX, Haas FM, Maechler H, Pierer G et al. (2003) Retrograde arterialized venous flap: an experimental study. Microsurgery 23: 130-134. doi:10.1002/micr.10108. PubMed: 12740885. 12740885

[21] Mutaf M, Tasaki Y, Fujii T (1998) Expansion of venous flaps: an experimental study in rats. Br J Plast Surg 51: 393-401. doi:10.1054/bjps.1997.0151. PubMed: 9771368. 9771368

[22] Woo SH, Kim SE, Lee TH, Jeong JH, Seul JH (1998) Effects of blood flow and venous network on the survival of the arterialized venous flap. Plast Reconstr Surg 101: 1280-1289. doi:10.1097/00006534-199804010-00019. PubMed: 9529214. 9529214

[23] Lenoble E, Foucher G, Voisin MC, Maurel A, Goutallier D (1993) Observations on experimental flow-through venous flaps. Br J Plast Surg 46: 378-383. doi:10.1016/0007-1226(93)90043-B. PubMed: 8369875. 8369875

[24] Gençosmanoğlu R, Ulgen O, Yaman C, Songür E, Akin Y et al. (1993) Mechanisms of viability in rabbit flank venous flaps. Ann Plast Surg 30: 60-66. doi:10.1097/00000637-199301000-00009. PubMed: 8333688. 8333688

[25] Yuan R, Shan Y, Zhu S (1998) Circulating mechanism of the "pure" venous flap: direct observation of microcirculation. J Reconstr Microsurg 14: 147-152. doi:10.1055/s-2007-1000158. PubMed: 9590608. 9590608

[26] Wong MS, Erdmann D, Sweis R, Pöllmann C, Farrar M et al. (2004) Basic fibroblast growth factor expression following surgical delay of rat transverse rectus abdominis myocutaneous flaps. Plast Reconstr Surg 113: 2030-2036. doi:10.1097/01.PRS.0000122217.16985.52. PubMed: 15253193. 15253193

[27] Matsushita K, Firrell JC, Ogden L, Tsai TM (1993) Blood flow and tissue survival in the rabbit venous flap. Plast Reconstr Surg 91: 127-136; discussion: 8416517. 8416517

[28] Lubiatowski P, Goldman CK, Gurunluoglu R, Carnevale K, Siemionow M (2002) Enhancement of epigastric skin flap survival by adenovirus-mediated VEGF gene therapy. Plast Reconstr Surg 109: 1986-1993. doi:10.1097/00006534-200205000-00031. PubMed: 11994603. 11994603

[29] Akdemir O, Hede Y, Zhang F, Lineaweaver WC, Arslan Z et al. (2011) Effects of taurine on reperfusion injury. J Plast Reconstr Aesthet Surg 64: 921-928. doi:10.1016/j.bjps.2010.12.007. PubMed: 21256822. 21256822

[30] Tao Y, Hu S, Lui KW, Chen S, Tang M (2011) Quantitative regression analysis of the cutaneous vascular territories in a rat model. Surg Radiol Anat 33: 789-799. doi:10.1007/s00276-011-0809-7. PubMed: 21455836. 21455836

[31] Chavoin JP, Rouge D, Vachaud M, Boccalon H, Costagliola M (1987) Island flaps with an exclusively venous pedicle. A report of eleven cases and a preliminary haemodynamic study. Br J Plast Surg 40: 149-154. doi:10.1016/0007-1226(87)90187-1. PubMed: 3567447. 3567447

[32] Baek SM, Weinberg H, Song Y, Park CG, Biller HF (1985) Experimental studies in the survival of venous island flaps without arterial inflow. Plast Reconstr Surg 75: 88-95. doi:10.1097/00006534-198501000-00020. PubMed: 3966113. 3966113

[33] Kovács AF (1998) Comparison of two types of arterialized venous forearm flaps for oral reconstruction and proposal of a reliable procedure. J Cranio-Maxillofac Surg 26: 249-254. doi:10.1016/S1010-5182(98)80021-8. PubMed: 9777504. 9777504

[34] Cho BC, Lee MS, Lee JH, Byun JS, Baik BS (1998) The effects of surgical and chemical delay procedures on the survival of arterialized venous flaps in rabbits. Plast Reconstr Surg 102: 1134-1143. doi:10.1097/00006534-199809040-00033. PubMed: 9734433. 9734433

[35] Byun JS, Constantinescu MA, Lee WP, May JW Jr. (1995) Effects of delay procedures on vasculature and survival of arterialized venous flaps: an experimental study in rabbits. Plast Reconstr Surg 96: 1650-1659. doi:10.1097/00006534-199512000-00019. PubMed: 7480285. 7480285

[36] Yan H, Brooks D, Jackson WD, Angel MF, Akdemir O et al. (2010) Improvement of prearterialized venous flap survival with delay procedure in rats. J Reconstr Microsurg 26: 193-200. doi:10.1055/s-0030-1247715. PubMed: 20119898. 20119898

[37] Suzuki Y, Suzuki K, Ishikawa K (1994) Direct monitoring of the microcirculation in experimental venous flaps with afferent arteriovenous fistulas. Br J Plast Surg 47: 554-559. doi:10.1016/0007-1226(94)90139-2. PubMed: 7697283. 7697283

[38] Setälä LP, Korvenoja EM, Härmä MA, Alhava EM, Uusaro AV et al. (2004) Glucose, lactate, and pyruvate response in an experimental model of microvascular flap ischemia and reperfusion: a microdialysis study. Microsurgery 24: 223-231. doi:10.1002/micr.20045. PubMed: 15160382. 15160382

[39] Setala L, Gudaviciene D (2013) Glucose and Lactate Metabolism in Well-Perfused and Compromised Microvascular Flaps. J Reconstr Microsurg, 29: 505–10. PubMed: 23757155. 23757155 10.1055/s-0033-1348039

[40] Su CT, Im MJ, Hoopes JE (1982) Tissue glucose and lactate following vascular occlusion in island skin flaps. Plast Reconstr Surg 70: 202-205. doi:10.1097/00006534-198208000-00014. PubMed: 7048369. 7048369

